# Does viral load alter behavior of the bee parasite *Varroa destructor*?

**DOI:** 10.1371/journal.pone.0217975

**Published:** 2019-06-13

**Authors:** Carl Giuffre, Sharon R. Lubkin, David R. Tarpy

**Affiliations:** 1 Department of Mathematics and Computer Sciences, St. Mary’s College of Southern Maryland, St. Mary’s City, Maryland, United States of America; 2 Department of Mathematics, North Carolina State University, Raleigh, North Carolina, United States of America; 3 Department of Entomology and Plant Pathology, North Carolina State University, Raleigh, North Carolina, United States of America; 4 W.M. Keck Center for Behavioral Biology, North Carolina State University, Raleigh, North Carolina, United States of America; University of North Carolina at Greensboro, UNITED STATES

## Abstract

The invasive mite *Varroa destructor* has negatively impacted global apiculture, by being a vector for many viruses of the honey bee (*Apis mellifera*). Until now, most studies have been limited to varroa-honey bee or virus-honey bee interactions. The aim of this study is to bridge the important research gap of varroa-virus interactions by correlating varroa behavior with viral load. Ten-minute video recordings of 200 varroa mites were analyzed, and average speeds of the mites were compared to individual qPCR viral loads for deformed wing virus (DWV) and sacbrood virus (SBV). Statistically significant models reveal that colony, DWV, and SBV all might play a role in mite behavior, suggesting that the varroa-virus interaction needs to be an integral part of future studies on honey bee pathogens.

## Introduction

The invasive mite *Varroa destructor* has negatively impacted apiculture worldwide [1]. Varroa experienced an evolutionary host-shift from the Asian honey bee (*Apis cerana*) to the European honey bee (*Apis mellifera*) as early as 1960 and has been strongly implicated for playing a role in Colony Collapse Disorder and reduced health of bees in general [1,2]. These ectoparasites go through two major phases in their life cycle—the reproductive and phoretic stages. During the reproductive stage, a single varroa female infests the cell of an immature honey bee (pupa), feeding on the hemolymph of the developing bee. In doing so, the parasite can directly vector several viruses within honey bee colonies [2–4]. During the phoretic stage, varroa mites emerge with the enclosed bee and continue to feed on adult honey bee hemolymph for sustenance [2] and continue to spread viral pathogens horizontally among nestmates [5]. Honey bee colonies can exceed 50,000 bees, with only one female reproductive (the queen) [2]. When a queen is infected with viral pathogens, the health of the entire colony can be compromised as she then has the potential to vertically transmit virus to her offspring through oviposition [5].

The Vector Manipulation Hypothesis suggests that pathogens can modify the motility behavior or host preference for vector organisms, aiding in the spread of pathogens to the target hosts [5]. There are many known examples in arthropods of viruses or parasites altering vector behavior [5–7]. The Vector Manipulation Hypothesis supports the possibility that infected mite vectors could exhibit higher motility than uninfected individuals. Such behavioral modifications could determine the success or failure for certain viruses to spread within and among honey bee colonies, and it could have significant consequences for global bee health and the means to mitigate disease.

The study of varroa is important for the future success of honey bee management. The introduction of varroa to the United States has heavily impacted colony health, honey bee economics, and integrated pest management [1,8,9]. The mode of action for many pesticides is similar for both insects and arachnids, both belonging to the same phylum, Arthropoda [10]. Thus, treating for varroa can have negative implications on honey bee colony health, even leading to mortality within or of the colony [10]. On the other hand, mites themselves impact colony health by vectoring pathogens [11]. This tradeoff forces beekeepers to make difficult decisions, and optimal varroa treatment strategy is not always clear.

As a further complication, varroa have developed resistance to common acaricide treatments [8,9], which has influenced modern studies emphasizing behavioral treatments over chemical ones. Since the introduction of varroa to honey bees has presumably modified bee behavior, most previous studies focus on how honey bees behave toward varroa or bee grooming [12–14]. Most of these assays examine some indirect artifact of honey bee behavior, such as the freeze-brood and sticky-board assays. One assay more directly measured the ability for a honey bee to bite and damage the exoskeleton or legs of varroa, rendering them dead or useless [15].

More than 18 honey bee viruses have been identified, with six major viruses at the center of global scientific interest: deformed wing virus, sacbrood virus, black queen cell virus, Kashmir bee virus, acute bee paralysis virus, and chronic bee paralysis virus [3,16,17]. We here focus on two of the more common and economically important viruses in honey bees: deformed wing virus (DWV) and sacbrood virus (SBV). DWV has been of scientific interest because of its connection with varroa transmission. Although DWV can be spread to larvae by vectored mites, physiological differences in infected individuals are not apparent until the adult stage of the honey bee [16–19]. The host bee pupates and often develops with deformed wings, rendering adults unable to contribute to foraging duties in the colony [16]. SBV targets the brood cycle of honey bees, preventing a brood from pupating and thus resulting in larval death. Both these viruses have been found in varroa, though it appears varroa are only vectors for DWV and not SBV [3,16]. The low mortality and virulence of DWV benefits both virus prevalence and varroa dispersal, whereas SBV increases the chance of varroa mortality alongside the dead honey bee brood [20]. The aim of this study is to test the Vector Manipulation Hypothesis by correlating mite behavioral phenotypes to their viral status, identifying the role this interaction plays in the entire honey bee system.

## Methods

### Mite collection

Mites were collected at the Lake Wheeler Honey Bee Research facility in Raleigh, NC. Once varroa infestations were identified in a colony, mites were gathered using the sugar-shake method, a process that safely dislodges live mites off their honey bee hosts [21]. The mites were subsequently gently rinsed in phosphate-buffered solution, removing excess sugar from their exoskeleton [21]. Mites were then placed in a 60 mm-diameter petri dish, creating a small arena for the mite to explore over the course of the experiment. Fifty mites were gathered from each of four unrelated colonies, yielding 200 experimental subjects.

### Video recording

**Fig 1:**
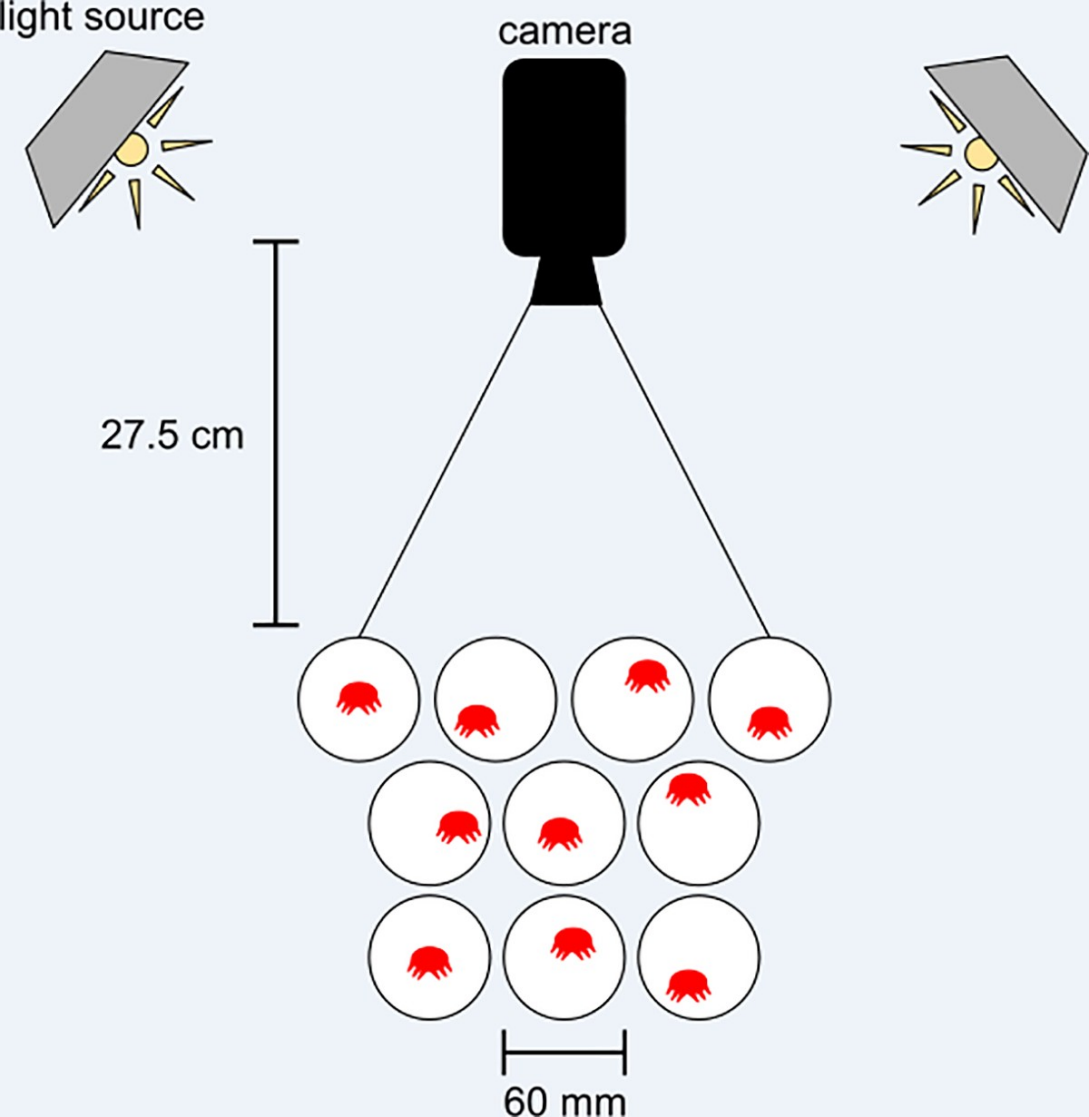
Experimental setup. A camera was mounted 27 cm directly above a collection of 10 petri dishes, with one varroa mite per dish. Two utility lamps were mounted in a square surrounding the recording area, angled to minimize glare off the dish and maximize illumination of the recording area (figure not to scale). Once the footage was recorded, mites were individually transferred to micropipette tubes and placed in a -80°C freezer for subsequent analysis of viral loads.

All recordings were taken in a dark room with one Sony Handycam mounted approximately 27 centimeters above the dishes containing the mites (Fig 1). Two utility light clamps (Coleman) were installed with 10W (60W-equivalent) LED light bulbs (Lighting Science) and placed on the perimeter of the recording arena to provide uniform lighting. Beneath the dishes was a sheet of white paper. Individual mites were recorded for 10 minutes at 30 frames per second with 1 minute of buffering at the beginning of each video, for a total recording time of 11 minutes. A total of 40 videos were recorded over the span of three days, with ten dishes per recording.

### Quantitative PCR

RNA was extracted from individual varroa mites [3] using the BioBasic EZ-10 Spin Column Total RNA Mini-Preps Kit, resuspended in water, and tested for β-actin and Apo28s as reference genes [22] and for the same viral targets via real-time PCR on the same machine using KAPA SYBR FAST One-Step qRT-PCR ABIPrism Kit. The reaction mix contained 2.5 μL of SYBR, 0.25 μL of primer, 0.2 μL of KAPA RT Mix, 1.25 μL of water and 1μL of sample for a final reaction volume of 5.2 μL. The qPCR program ran at 42°C for 5 min, 52°C for 3 min, 95°C for 3 min, then cycled 40 times through 95°C for 3 sec, 58°C for 25 sec, and 72°C for 1 sec, then performed a melt curve step. Results were verified by melt curve temperature and normalized [23] via GeNorm.

### Video analysis

**Fig 2:**
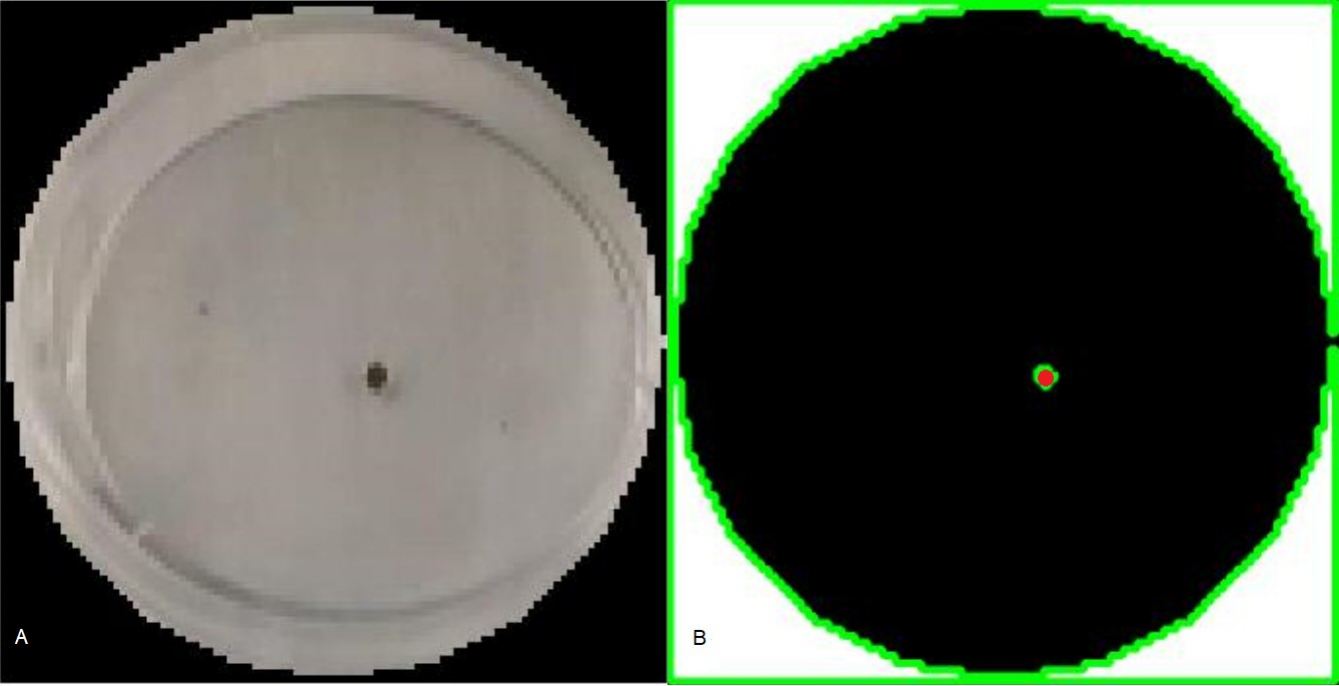
Visualization of the steps for the mite detection algorithm. For each video frame, (A) Petri dish edges were determined then (B) the images were converted to grayscale and binary images were produced using a threshold value of 0.255 on a 0–1 scale. Finally, the convex hull of the mite was used to compute a centroid in the Cartesian plane.

Videos were processed using a custom algorithm written in MATLAB R2105b (The Math Works Inc., Natick, Massachusetts, United States). This algorithm follows four steps, requiring minimal user intervention only on the first step: (1) Dish partitioning, (2) Frame extraction and thresholding, (3) Centroid calculation, and (4) Metric calculation. Steps 1–3 of the algorithm are visualized in Fig 2.

Because multiple dishes were filmed simultaneously, individual dishes had to be identified and segmented from the videos. The manual partitioning step forces the user to define boundaries on each dish using the first frame from the video and the MATLAB R2015b Image Processing Toolbox command *imcircle*. This step serves a dual purpose in the context of this experiment. First, it enables the centroids calculated in step 3 to be joined and identified as belonging to the same varroa mite, then centered at the origin, irrespective of where the dish was placed in the recording area. Second, it allows for quick conversion between pixel and metric data, using the diameter of the petri dish as a scale.

Video frames were converted to grayscale. ImageJ (National Institutes of Health, Bethesda MD–USA) was used to estimate the proper binary thresholding value for the mites (65 of 255 or ≈ 0.26, 255 = white), which was applied uniformly across every image sequence. It was important to ensure that the thresholding value was low enough to distinguish the mite from the background, but high enough to avoid tracking shadows cast by mites or dish edges. The result was a discrete sequence of images containing an isolated cluster of pixels, representative of a single varroa mite. On occasion, pixel values were misinterpreted in the thresholding step. The MATLAB command *regionprops* (The Math Works Inc., Natick, Massachusetts, United States) computed the convex hull for candidate clusters of pixels that may have been the mite. If the cluster size was lower than the expected pixel area of a mite, it was eliminated as background noise. The centroid of the remaining pixel cluster was then interpreted as the Euclidean coordinate location of the mite, which was used to determine behavioral parameters that might be of interest.

Standard measurements—such as velocity and average speed—are useful in analyzing complete, continuous, and smooth data. However, recorded mite tracks appeared to be discontinuous at some points for the following reason. Varroa are extremely flat organisms, and therefore they appear as an ellipse when viewed dorsally but only as a sliver when viewed laterally. Since any mite can be hard to detect if it turns edge-on, careful consideration had to be made regarding how behavioral metrics were calculated in the presence of discontinuities in mite tracks. Furthermore, centroid calculation can introduce small perturbations in coordinate data. These perturbations improperly skewed typical rate-of-change calculations such as instantaneous speed and velocity.

**Fig 3:**
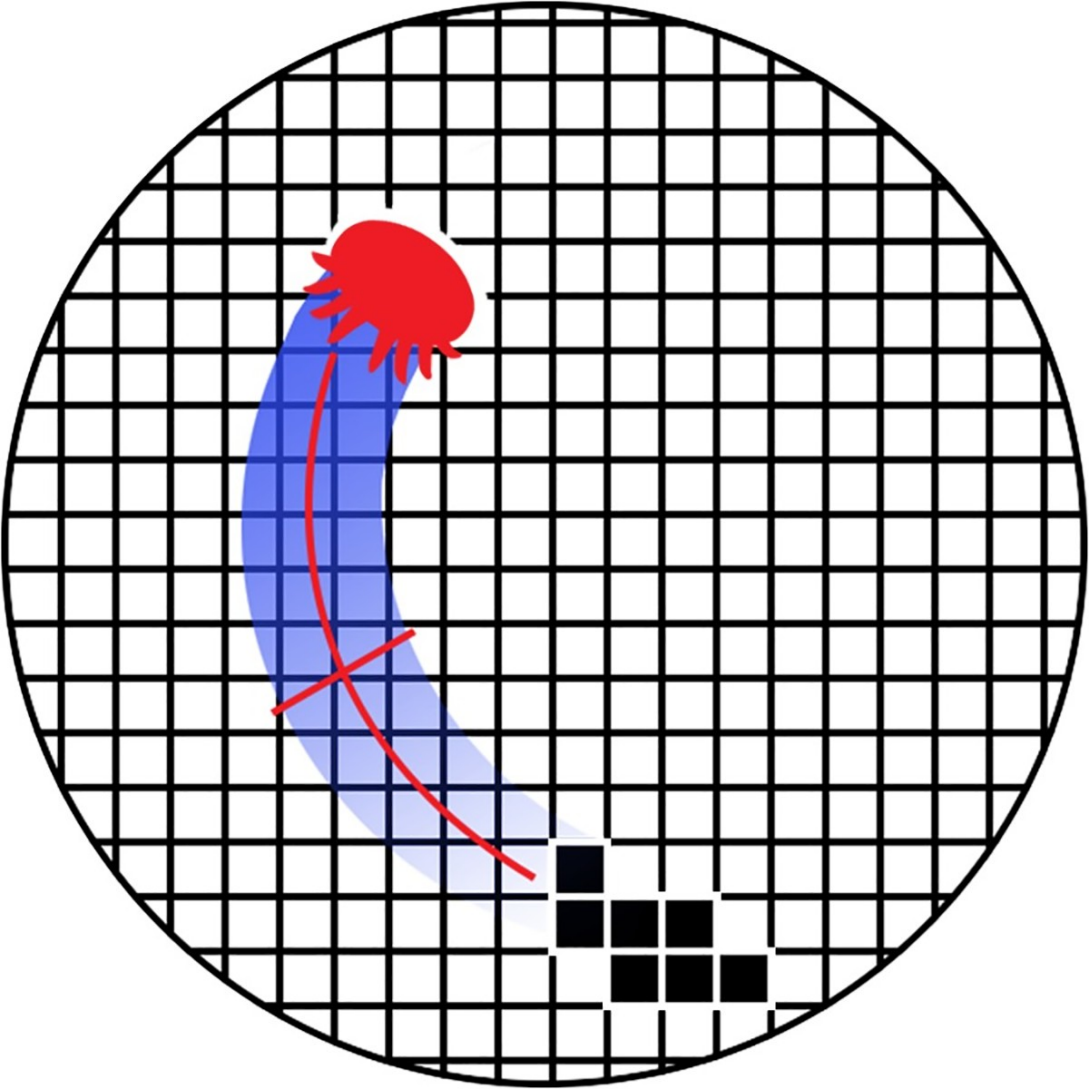
Cartoon for average speed metric. The petri dish image space was gridded with 1 mm by 1 mm squares, corresponding to the typical width of a varroa mite. Grid positions containing a mite centroid are assigned a value of 1, otherwise 0. Average speed is calculated from the fraction of positions filled divided by the recording time.

To compensate, a surrogate metric for average speed was calculated in step 4 of the processing algorithm. Because the mites are approximately 1 mm in width, dividing the total track area covered by a mite per unit time by the mite width yields a reasonable estimate of the mite average speed. Each dish was gridded with 1 mm^2^ squares and treated as a binary 60 by 60 “visitation matrix” (Fig 3). If a mite centroid was found in any given grid square, it was assigned a value of one, otherwise zero. The total area traveled, in square millimeters, could then be divided by the amount of time the mite was successfully tracked, resulting in the following estimate of average speed *S*:

$$S\;(mm/min)=\frac{\left(number\;of\;1\;mm^{2}\;squares\right)}{\left(total\;time\;tracked\;in\;mins\right)}\times \frac{1}{1\;mm}$$ (1)

All data files used in this study, including raw video footage, qPCR, and mite metric data, are freely available, and can be found at https://osf.io/ehdr3/.

## Results

**Fig 4:**
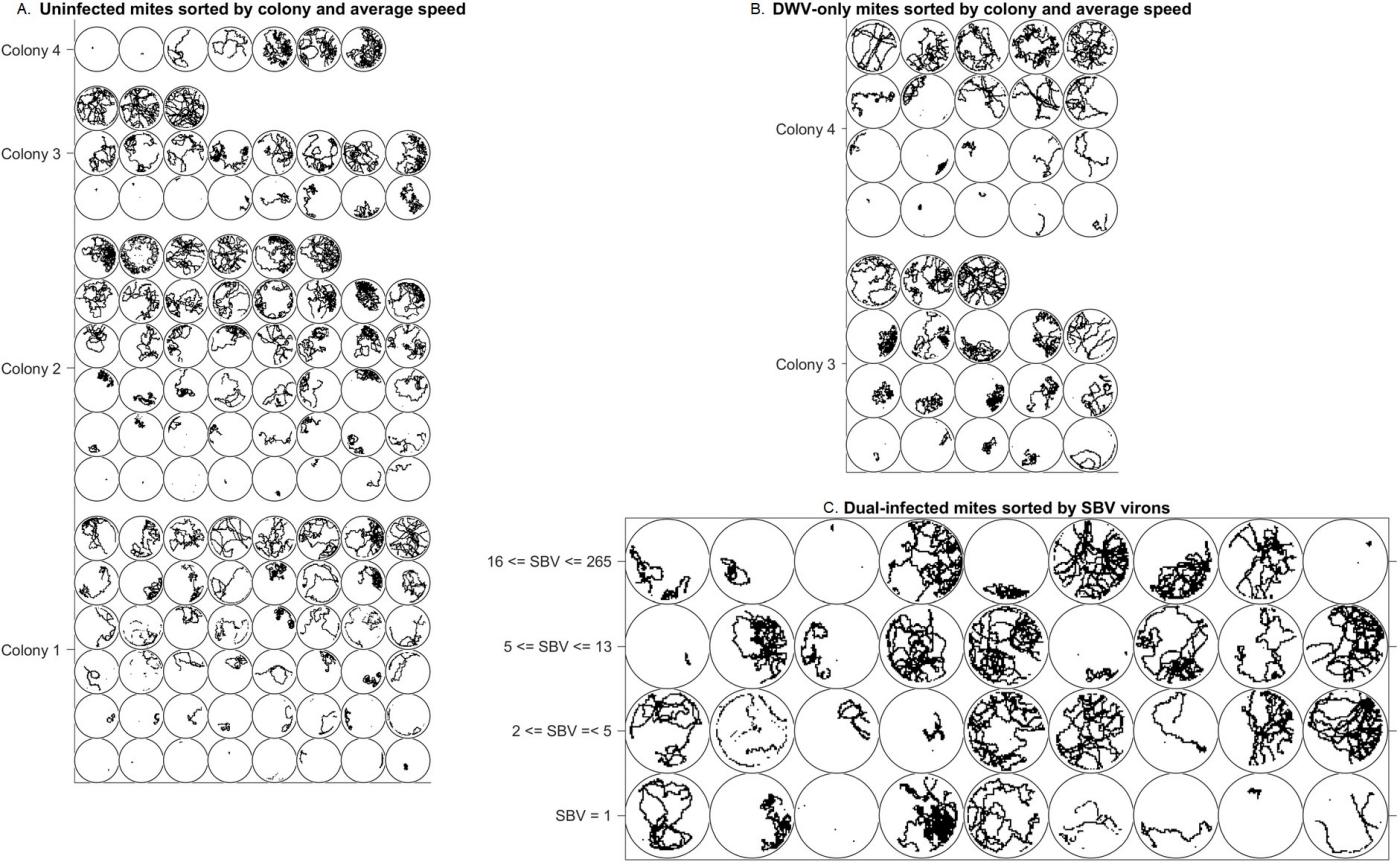
Visualization of mite tracks. Mite tracks grouped by colony and viral status. (A) Uninfected mites grouped by colony and sorted by average speed. (B) Mites only infected by DWV, grouped by colony and sorted by average speed. (C) Mites infected with both DWV and SBV, sorted by SBV viron count.

The behavioral and qPCR data were statistically analyzed using JMP Pro 11.0 (SAS Institute, Cary, NC, USA). Mites were tested for a suite of seven common honey bee viruses [24] although the collected mites only tested positive for DWV and SBV. Of the 200 total mites, 194 remained for statistical analysis after accounting for various technical errors or small representation in categories (Table 1). Of those 194, whose tracks are displayed in Fig 4A–4C, 120 tested negative for infection (Fig 4A). Colonies 1 and 2 were primarily uninfected, with only two mites from each colony testing positive for both DWV and SBV. In contrast, colonies 3 and 4 displayed a variety of infection patterns: uninfected (Fig 4A), infected with DWV only (Fig 4B), and infected with both DWV and SBV (Fig 4C).

**Table 1:** Breakdown of processed data set and exclusions.

colony	analyzed			not analyzed			total
	uninfected	infected		SBV only	qPCR failure	video failure	
		DWV	both				
1	48	0	2	0	0	0	50
2	46	0	2	0	0	2	50
3	19	18	10	0	3	0	50
4	7	20	22	1	0	0	50
**totals**	120	38	36	1	3	2	200

Of 200 mites, 6 were excluded from statistical analysis: 1 mite was only infected by SBV, 3 mites were not analyzed by qPCR, and 2 mites could not be tracked for any portion of the ten-minute video. Infection types for the remaining 194 mites (uninfected/DWV-only/both) are also provided.

We tested a variety of statistical models using the input variables {colony ID, kDWV, SBV} to predict the response variable AS (average speed, mm/min, determined from video analysis). SBV were in units of virions, and kDWV were in units of kilovirions. The general form of the statistical model used was

AverageSpeed(AS)=α(intercept)+β*Colony+γ*kDWV+δ*SBV+θ*(kDWV−D0)*(SBV−S0)+error (2)

**Fig 5:**
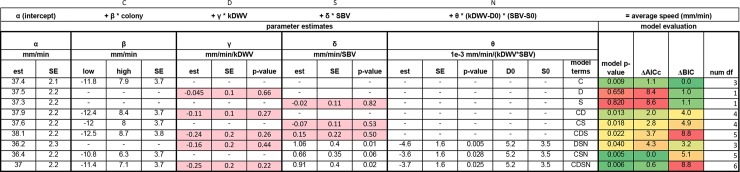
Statistical models. Models were combinatorially tested across multiple levels. The leading row of the table provides quick identification for the model and parameters of interest for that model (i.e., Model CD uses parameters α, β, and γ). Poor parameter estimates are highlighted (pink). AICc and BIC metrics were normalized according to the expression *ΔIC = IC/max(IC)– 1*, then color-coded on a scale from 0 (green, strong information criterion model selection) to *max(ΔIC)* (red, unfavorable information criterion model selection) so that model selection could be quickly verified. Models with *Δ*IC = 0 are boldfaced. Additional rejected models (p > .05) not shown.

Where *α*, *β*, *γ*, *δ*, *θ*, *D0*, and *S0* are free fitting parameters in the appropriate units. This general model form includes model terms for intercept, colony ID (C), kDWV (D), SBV (S), and a nonlinear interaction (N) between viruses. We combinatorially tested models containing or omitting each of these model terms, e.g. Model CD contains only the intercept, colony, and kDWV dependence (thus setting parameters *β*, *δ*, and *θ* to 0). Additionally, we tested models with nonlinear dependence on kDWV or SBV (not shown). In general, models were rejected at a level of α = 0.05. Parameter fits for the simplest models are shown in Fig 5. Additional rejected models are not shown.

Colony effects are not the major effects of interest in this study. However, it was important to establish whether colony-level effects were present before building up more complicated models. Model C was accepted with a p-value < 0.01 (numerator d.f. = 3), indicating that the colony of origin influences average speed of the mite.

The main effects of viral loads (kDWV, SBV) on average speed were explored in Models D and S, ignoring colony-level effects. Model D (p = 0.66, d.f. = 1) and Model S (p = 0.82, d.f. = 1) were both rejected. However, addition of the nonlinear viral interaction yielded Model DSN, which was accepted with a p-value of 0.04 (d.f. = 3). Finally, Model CDS (p = 0.02, d.f. = 5) and Model CDSN (p < 0.01, d.f. = 6) were both accepted.

Selection of appropriate and parsimonious statistical models was done using Bayesian information criterion (BIC) and corrected Akaike information criterion (AICc), two philosophically different approaches for model selection. Both metrics attempt to maximize goodness-of-fit, while minimizing the number of parameters used to avoid overfitting the data. Model C had the smallest BIC, and Model CSN had the smallest AICc. BIC and AICc are reported in Fig 5.

## Discussion

BIC favors selection of Model C (smallest BIC), which suggests that mite viral load seems to be unrelated to mite behavior. At the colony level, phoretic mites may modify their behavior based on environmental conditions, such as bee population, brood availability, hive temperature, or even bee grooming. Although none of these variables were measured in the current study, environmental conditions clearly play a direct role in virus’ ability to spread, by impacting vector motility. It could be argued that colonies have different viral profiles responsible for these global effects. Unfortunately, four colonies were not sufficient to determine such effects.

AICc selects Model CSN, which also has the lowest p-value. The estimates of γ (mm/min/kDWV) have high variability between models, are nearly centered at zero, and have high p-values, bringing to question the kind of impact DWV has on mite behavior. However, the selection of statistical models that include cross-terms without also including the related linear terms has been a topic of debate in the statistical community [25].

The most complicated Model CDSN also performs very well, but its AICc is higher than Model CSN's AICc. Nonetheless, Model CDSN is of interest, because while DWV is known to replicate in varroa, SBV is not [3,16]. SBV should not be modifying varroa RNA, and therefore, should have no interactive relationship with their behavior [3]. One possible explanation for this might be that SBV plays a more important role in colony-level effects, which indirectly changes their behavior, however the Model CS(C*S) with colony and SBV crossed was rejected at level α = 0.05. Recent studies show that viruses can interact with one another in nontrivial ways, including altered host susceptibility due to a breakdown in physical barriers in both plants and animals, which may be true for vectors, as well [26–28]. Although SBV does not modify varroa RNA, it could be that SBV-bearing mites are more susceptible to effects from DWV, or that SBV replication occurs in the presence of DWV, but this possibility will require further study. Our data indicate that the DWV-SBV interaction generally causes mites to move more slowly. SBV seems is correlated with faster mite movement, except in Models S, CS and CDS, where the p-values for δ (mm/min/SBV) estimation are high (Fig 5).

Many questions remain, such as how or why DWV and SBV interact. This study had mite selection and tracking performed blind (*a priori*) to the types and levels of infection: it was possible that none of the mites were infected, or that they had a suite of viruses with even more complicated levels of cross-infection. The limited results of this study suggest the need for a more controlled experiment, where varroa are inoculated with specific virus levels and types, or where varroa are gathered from more than four colonies over a longer timeframe, with greater knowledge of colony phenotypes or environments.

Varroa mites not only vector honey bee-specific viruses, but they also carry their own viruses that do not infect *A*. *mellifera* [29–31]. The role that these varroa-specific viruses play on varroa movement and behavior is still unknown, and how they potentially interact with economically important bee viruses remains unclear. Both of these avenues warrant further empirical research and are intriguing to fully understand varroa as a noxious parasite of honey bee colonies worldwide. However, because varroa-specific pathogens would not be selectively favored to alter the behavior of their vectors to increase infection rates of their main host, they are unlikely to have significant bearing on the Vector Manipulation Hypothesis, and thus, were not measured as part of this preliminary study in lieu of focusing on the honey bee-specific viruses (DWV and SBV). Based on our preliminary findings here, it is clear that additional research is warranted both in vitro as well as in vivo to focus on possible behavioral changes of varroa mites as pathogenic vectors.

This assay is a novel attempt at exploring varroa behavior, in addition to tying results to the Vector Manipulation Hypothesis. The AICc values for all models suggest that viral loads seem to play an important role in how varroa explore their surroundings, supporting this hypothesis and akin to findings in other areas of entomology [5,6]. Varroa behavior should be studied more for the role that it plays in the host-vector-disease interaction, and this study provides a framework for such future studies.

The bioassay developed in this study attempts to facilitate data gathering by making it high-throughput and easy-to-track. It could be modified, e.g. by using particle image velocimetry, or restricting the mite to the upper or lower surface of the dish. Moreover, this study opens the door for a host-preference study on varroa. Honey bee pheromones are notably vital for communication in the dark environment of a hive [2]. Using a similar experimental design, one could test viral effects on host preference for *A*. *mellifera* drones, versus workers, versus *A*. *cerana*. Perhaps mite viral ethology could better explain the evolutionary jump from *A*. *cerana* to *A*. *mellifera*, or further support it. Testing and measuring mite behavior in vitro may help untangle the more complicated vector-disease dynamics of the hive.
